# Effectiveness and safety of an ayurvedic nutraceutical for improving clinical parameters of liver function in participants with metabolic dysfunction-associated steatotic liver disease: a randomized, placebo-controlled, preliminary phase 2 exploratory clinical trial

**DOI:** 10.3389/fnut.2026.1761666

**Published:** 2026-03-12

**Authors:** Helen Messier, Jignesh Patel, Vinay Bhomia, Dilip Kataria, Ghanashyam Patel

**Affiliations:** 1 Altum Medical, Orlando, FL, United States; 2 Sanjivani Hospital, Gujarat, India; 3 Green Medic Solution, Gujarat, India

**Keywords:** dandelion, fatty liver grade, ginger, metabolic dysfunction-associated steatohepatitis, milk thistle, oral nutraceutical, turmeric

## Abstract

**Introduction:**

Metabolic dysfunction-associated steatotic liver disease (MASLD) lacks treatment options, with few evidence-based choices beyond lifestyle modification. This is the first clinical study to assess the efficacy of an organic liquid oral nutraceutical composed of turmeric extract, dandelion powder, milk thistle extract, and ginger powder in MASLD.

**Methods:**

This was a randomized, double-blind, placebo-controlled, preliminary phase 2 exploratory trial conducted at a single center (Sanjivani Super Speciality Hospitals Pvt. Ltd., Gujarat, India) between September 7, 2021 and November 25, 2021. Individuals aged 18–70 years and diagnosed with fatty liver (grades 1–3) were eligible to participate. Participants were randomized 1:1 to receive the test product or placebo, which were both provided as a 60 mL oral solution to be consumed twice daily with a meal for 60 days. The primary endpoint was proportion of participants with improvement in ultrasonography-based fatty liver grading from baseline to Day 60.

**Results:**

Fifteen patients each were enrolled in the test product and placebo groups. The mean [standard deviation (SD)] age was 40.6 (10.8) years and 83.3% were male. A significantly higher proportion of patients in the nutraceutical product group had fatty liver grade improvement at Day 60 vs. placebo [60.0% (9/15) vs. 13.3% (2/15); *p* = 0.008]. Mean (SD) change in fatty liver grading was −0.60 (0.51) and −0.13 (0.35) for test product vs. placebo (*p* = 0.007). Adverse events were mild with similar incidence in both groups. The results are limited by the small sample size, single-center design, and short study duration.

**Discussion:**

This study provides preliminary evidence that this organic nutraceutical may improve steatosis grade in individuals with MASLD.

**Clinical trial registration:**

Clinical Trial Registry of India (CTRI/2021/08/035359).

## 1 Introduction

Metabolic dysfunction-associated steatotic liver disease [MASLD; formerly known as non-alcoholic fatty liver disease (NAFLD)] is caused by chronic fat deposition in the liver and is the result of complex interactions involving metabolic dysregulation and insulin resistance (1). Imaging tests, including FibroScan, which estimates the amount of fat and fibrosis present in the liver, are used to look for signs of MASLD (2). Fatty liver grading (grades 0–3) is used to determine the extent of fat buildup in the liver, with higher grades indicating a greater amount of fat deposition in the liver. People who have confirmed hepatic steatosis (i.e., accumulation of excess fat in the liver) based on imaging tests and who meet at least one of the cardiometabolic criteria can be diagnosed with MASLD (1). Some patients with MASLD will have metabolic dysfunction-associated steatohepatitis (MASH) which is characterized by hepatocellular ballooning and lobular inflammation (3). It is estimated that around one-third of the global population has MASLD, and the prevalence is increasing in most countries (4). MASLD is associated with the development of cirrhosis, liver failure, liver cancer, and type 2 diabetes (5). The main treatment for MASLD is lifestyle modification (e.g., healthy diet, regular exercise), weight loss, and management of comorbid conditions, such as type 2 diabetes (6).

Several herbal extracts have demonstrated benefits for liver health. Among these are turmeric (*Curcuma longa*), dandelion (*Taraxacum officinale*), ginger (*Zingiber officinale*), and milk thistle (*Silybum marianum*). In particular, turmeric has been shown to promote healthy liver cell metabolism and support tissues exposed to oxidative stress, including liver (7). Dandelion aids in the metabolism of lipids and insulin (8) and ginger has antioxidant properties and supports healthy liver function (9, 10). Milk thistle has shown hepatoprotective properties via its antioxidant, anti-inflammatory, and antifibrotic mechanisms (11). Berberine, a bioactive plant compound, has demonstrated positive impacts on lipid parameters, hepatic markers, insulin resistance, and degree of steatosis in patients with MASLD (12). Studies have also investigated the effects of coenzyme Q10 supplementation, demonstrating improvements in liver and cardiac function, and reduced inflammation and fatty liver grade; however, its use may be limited by poor bioavailability and the need for high doses (13, 14). Vitamin E, a potent antioxidant, has been extensively studied and shown to reduce liver enzymes and improve histological parameters such as steatosis and inflammation in patients with MASLD, though its efficacy in improving fibrosis remains uncertain (15). Additionally, higher dietary choline intake has been inversely associated with MASLD risk in U.S. adults (16). Choline supplementation has demonstrated favorable effects on hepatic steatosis, oxidative stress, inflammatory markers, and lipid profiles, likely through its role in hepatic phospholipid synthesis and lipid export (17, 18). There is some evidence that omega 3 supplementation may be beneficial. Indeed, a clinical trial of flaxseed powder supplementation in patients with MASLD demonstrated improvements in lipid deposition, body composition indicators, liver function, and lipid metabolism after the 12-week intervention compared with baseline (19). However, in all cases, further study is needed to confirm any preliminary findings.

An organic nutraceutical liquid nutraceutical formulation containing a mix of herbal extracts (turmeric, dandelion, ginger, and milk thistle) has been shown to significantly improve liver function test values in a clinical trial of healthy adults who took the herbal product twice daily for 180 days. Specifically, there was greater improvement in levels of alanine aminotransferase [ALT], aspartate aminotransferase [AST], alanine phosphatase [ALP], and gamma-glutamyl transferase [GGT] from baseline to end of study with the nutraceutical product than placebo (20). An important feature of this product is that it is made using organic ingredients, avoiding pesticide residues that may be present in conventionally grown herbal supplements (21). Further, the product is available in a convenient, portable, liquid formulation containing the multiple herbal extracts. A potential additional benefit is that of a liquid formulation, which may enhance adherence compared to an equivalent dose in capsule form, which may lead to “capsule fatigue” (22, 23).

Considering the observed improvement in biochemical markers of liver function in healthy participants following daily supplementation with the nutraceutical product (20) and given the lack of alternative therapeutic options for MASLD, further study of this product in patients with liver disease is warranted. Therefore, this clinical study aimed to evaluate the efficacy and safety of the nutraceutical test product in participants with a fatty liver diagnosis.

## 2 Participants and methods

### 2.1 Participants

Individuals aged between 18 and 70 years and who were diagnosed with fatty liver (grades 1–3) based on ultrasonography were eligible to participate. The main exclusion criteria were a known or suspected hypersensitivity to any of the ingredients in the test product; fatty liver secondary to alcohol consumption; a history of regular alcohol consumption exceeding 14 drinks/week for female patients or 21 drinks/week for male patients within the 6 months prior to screening; an addiction to alcohol or drugs; a history or presence of coronary, renal, pulmonary, or thyroid disease; an AST or ALT greater than 5 × the upper limit of normal (ULN); serum bilirubin greater than the ULN and platelet count < 95,000/μL; use of hypolipidemic medications or any drug known to affect hepatic function in the 4 weeks prior to randomization; or a history of hypothyroidism, obstructive sleep apnea, total parenteral nutrition, short bowel syndrome, or pancreatoduodenal resection that were secondary causes of fatty liver.

Hyperlipidemic medications, which included statins, fibrates, bile acid sequestrants, niacin, ezetimibe, alirocumab, and evolocumab, were prohibited during the study.

The clinical trial protocol was approved by the Sanjivani Hospital Ethics Committee at Sanjivani Super Speciality Hospitals Pvt. Ltd., Gujarat, India. The study was conducted according to the principles outlined in the Declaration of Helsinki and the International Council for Harmonization Good Clinical Practice guidelines, and Good Clinical Practice Guidelines for Clinical Trials in Ayurveda, Siddha and Unani Medicine. All study participants provided written informed consent. The study was prospectively registered at the Clinical Trial Registry of India (CTRI/2021/08/035359); registered on March 8, 2021). Neither patients nor the public were involved in the design, conduct, or reporting of the trial.

### 2.2 Study design and treatments

This was a prospective, randomized, double-blind, placebo-controlled, parallel group, preliminary phase 2 exploratory clinical trial to evaluate the effectiveness and safety of an oral liquid mixed herbal nutraceutical. This study was conducted at a single center (Sanjivani Super Speciality Hospitals Pvt. Ltd., Gujarat, India, a multi-specialty hospital) between September 7, 2021 and November 25, 2021. Participants attended a screening visit within the 14 days prior to the study start where baseline clinical data and laboratory values were collected. Participants were then randomized 1:1 (Day 0) to receive either the test product 60 mL twice daily or placebo 60 mL twice daily for 60 days. Participants received a 30-day supply of the test product and a subject diary card. Follow-up visits were conducted on Day 31 (±2 days) where participants received an additional 30-day supply of the test product and a new subject diary card, and Day 61 (±2 days) which was the end of treatment (EOT) visit. Follow-up visits included a physical examination, laboratory investigations, assessment of subject diary card and study treatment compliance, and safety monitoring. A safety assessment was conducted on Day 67 (±2 days) via a telephone. Efficacy assessments included MASLD grading via ultrasonography, Controlled Attenuation Parameter (CAP) score via FibroScan^®^, and AST to Platelet Ratio Index (APRI) score which are non-invasive markers selected because of their established correlation with hepatic steatosis and fibrosis risk; lipid profile parameters (total cholesterol, high-density lipoproteins [HDL], low-density lipoproteins [LDL], and triglycerides); and liver function tests (ALT and AST). Adverse events (AEs) and serious AEs (SAEs) were monitored. AEs and SAEs were coded and classified using the Medical Dictionary for Regulatory Activities, version 24.1. Ultrasonography grading was performed by a single experienced radiologist blinded to treatment assignment to reduce inter-observer variability.

The test product (Dose for your Liver^®^ Eetho Brands Inc., Miami, Florida, USA) is a plant-based nutraceutical designed to support liver function. It contains the active ingredients organic turmeric extract, organic dandelion powder, organic ginger powder, and organic milk thistle extract. Both the test product and placebo contained reverse osmosis water, erythritol, citric acid, monk fruit extract, and natural orange flavoring. The study intervention and placebo were identical in appearance, taste, and packaging. Each serving of the test product or placebo was provided as a 60 mL oral solution and was to be consumed twice daily (morning and evening) with a meal for the 60-day treatment period. Participants were advised to maintain a simple, staple diet, though there were no strict dietary or exercise requirements during the study.

Block randomization code was generated with a ratio of 1:1 allocation for test product vs. placebo treatment using SAS software, version 9.4 (SAS Institute; Cary, NC, USA) by a biostatistician at Statiza Statistical Services, Ahmedabad, India. Participants and investigators were blinded to the intervention assignments. The biostatistician, who was independent of this study, securely maintained the blinding of randomization codes, ensuring the concealment of group assignments until the completion of the data analysis phase. Blinding envelopes containing the treatment allocations with randomization numbers were prepared by the biostatistician and distributed to each study site to be used if emergency unblinding was needed.

### 2.3 Study endpoints

The primary efficacy endpoint was the proportion of participants who showed improvement in ultrasonography-based fatty liver grading from baseline to Day 60. Secondary efficacy endpoints were a change from baseline to Day 60 in fatty liver grading, APRI, CAP score based on FibroScan^®^ assessment, and lipid profile and liver enzyme (ALT and AST) values.

### 2.4 Statistical analysis

This study was exploratory in nature, and no formal sample size calculation was performed. The 30 enrolled participants were considered sufficient to evaluate the efficacy and safety of the test product compared with placebo.

Continuous data were summarized using descriptive statistics and categorical variables were summarized using frequency and percent. The efficacy analyses were performed on the modified intent-to-treat (mITT) population, which included all randomized participants who received at least one dose of the investigational product and returned for the EOT visit. The safety analysis was performed on the safety population, which included all randomized participants who received at least one dose of the investigational product. For the efficacy endpoints, statistical significance was tested using a two-sample proportion test. Change in fatty liver grading from baseline to Day 60 was compared using paired *t*-tests for each treatment group and two sample *t*-tests for the between group comparisons. Change from baseline to Day 60 for the secondary endpoints was compared using paired *t*-tests for intragroup comparisons and a two-sample *t*-test for between group comparisons. Effect sizes (Hedges' g, to account for the small sample size) and corresponding 95% confidence intervals for the between-group differences were also calculated. Missing data were not imputed. A *p*-value of < 0.05 was considered statistically significant. Statistical analyses were performed using SAS version 9.4 (SAS Institute; Cary, NC, USA).

## 3 Results

### 3.1 Participants

**Figure 1:**
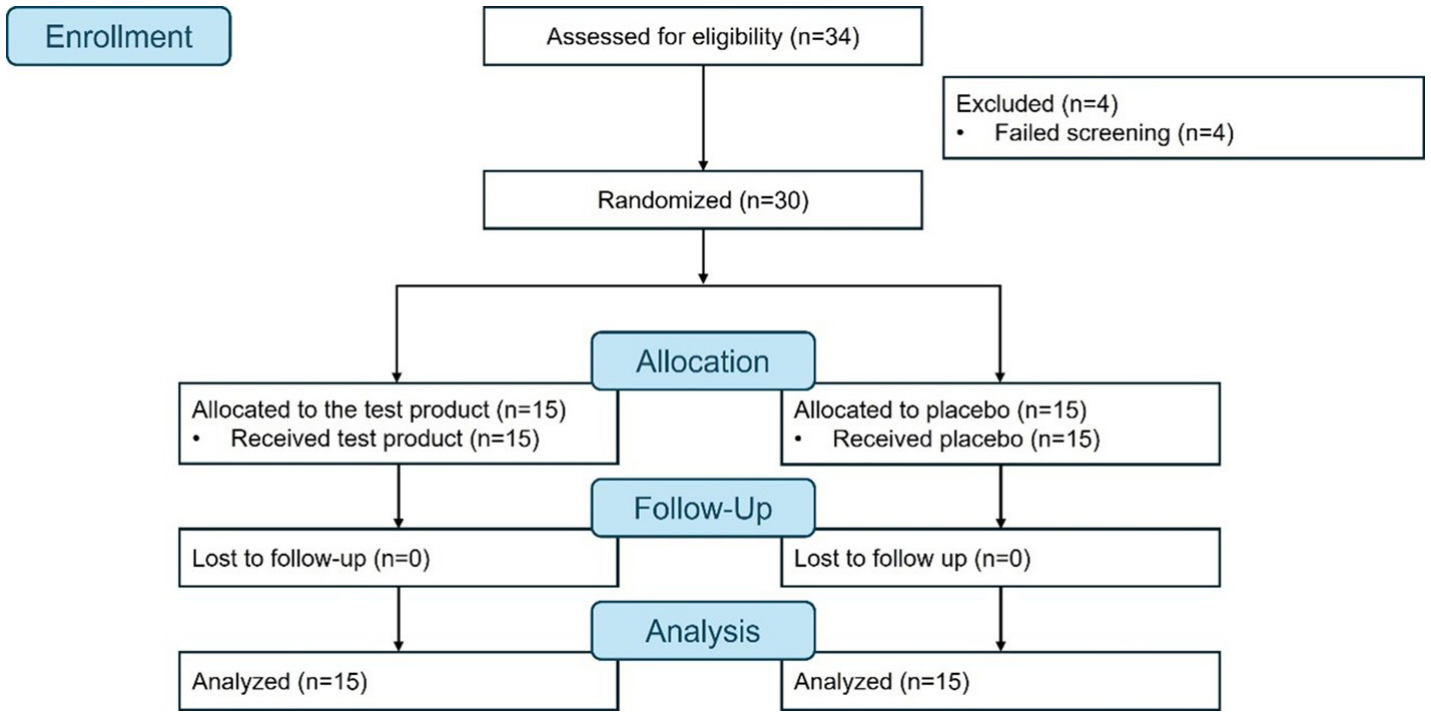
Flow diagram.

The first participant was enrolled on September 7, 2021, and the study was completed on November 25, 2021. A total of 34 participants were screened, four failed screening, and 30 were randomized and completed the study (test product, *n* = 15; placebo, *n* = 15) (Figure 1). All 15 participants attended all the scheduled study visits and were included in the efficacy and safety analyses.

In the test product and placebo groups, the respective mean [standard deviation (SD)] age was 39.3 (9.4) years and 41.8 (12.2) years, 73.3% (11/15) and 93.3% (14/15) of participants were male, and most had a fatty liver grade 2 [86.7% (13/15) and 73.3% (11/15)] (Table 1). Treatment compliance was reported for all participants; the mean (SD) number of total doses taken was 123 (2) for the test product group and 121 (3) for the placebo group.

**Table 1:** Participant demographic and clinical characteristics.

	**Test product (*N* = 15)**	**Placebo (*N* = 15)**
Age, years, mean (SD)	39.3 (9.4)	41.8 (12.2)
**Ethnicity**, ***n*** **(%)**		
Not Hispanic or Latino	15 (100)	15 (100)
**Sex**, ***n*** **(%)**		
Male	11 (73.3)	14 (93.3)
Female	4 (26.7)	1 (6.7)
**Race**, ***n*** **(%)**		
Asian	15 (100)	15 (100)
**Fatty liver grade**, ***n*** **(%)**		
Grade 1	2 (13.3)	4 (26.7)
Grade 2	13 (86.7)	11 (73.3)
Grade 3	0	0
**Liver health assessments, mean (SD)**		
APRI score	0.764 (0.150)	0.754 (0.152)
CAP score dB/m	274.5 (10.1)	268.9 (6.9)
**Lipids, mg/dL, mean (SD)**		
Total cholesterol	164.5 (13.0)	161.6 (10.4)
HDL	41.8 (4.9)	41.3 (4.7)
LDL	89.8 (21.2)	97.6 (15.8)
Triglycerides	142.2 (14.1)	141.1 (14.0)
**Liver function tests, mean (SD)**		
ALT, IU/L	70.3 (8.0)	68.7 (8.5)
AST, IU/L	62.0 (7.4)	58.9 (5.1)

ALT, alanine transaminase; APRI, AST to platelet ratio index; AST, aspartate aminotransferase; CAP, controlled attenuation parameter; HDL, high-density lipoprotein; LDL, low-density lipoprotein; SD, standard deviation.

### 3.2 Efficacy endpoints

**Figure 2:**
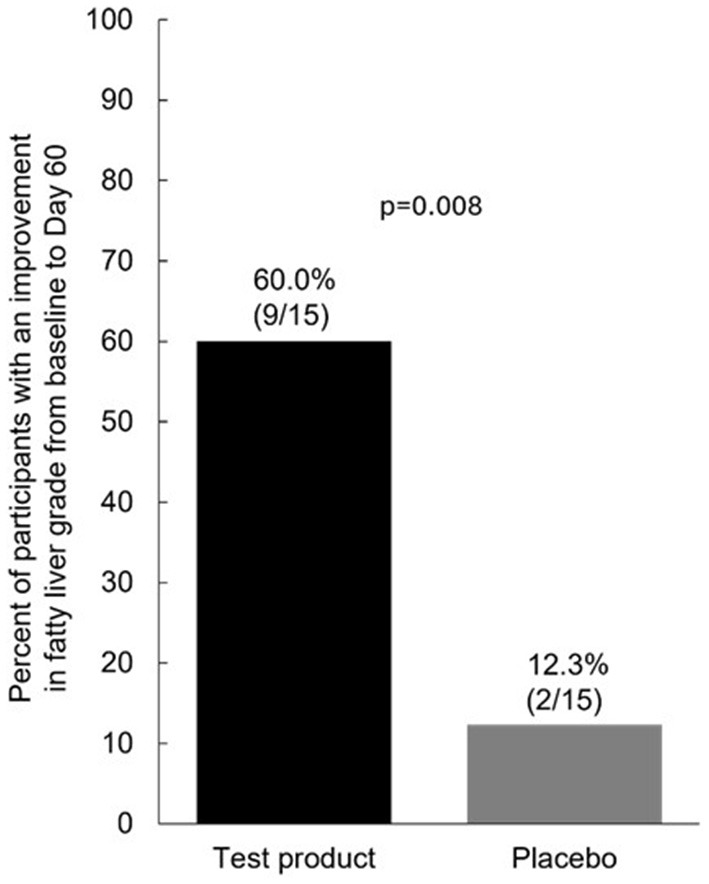
Percent of participants with an improvement in fatty liver grade from baseline to Day 60 *p*-values were calculated using a paired *t*-test.

**Figure 3:**
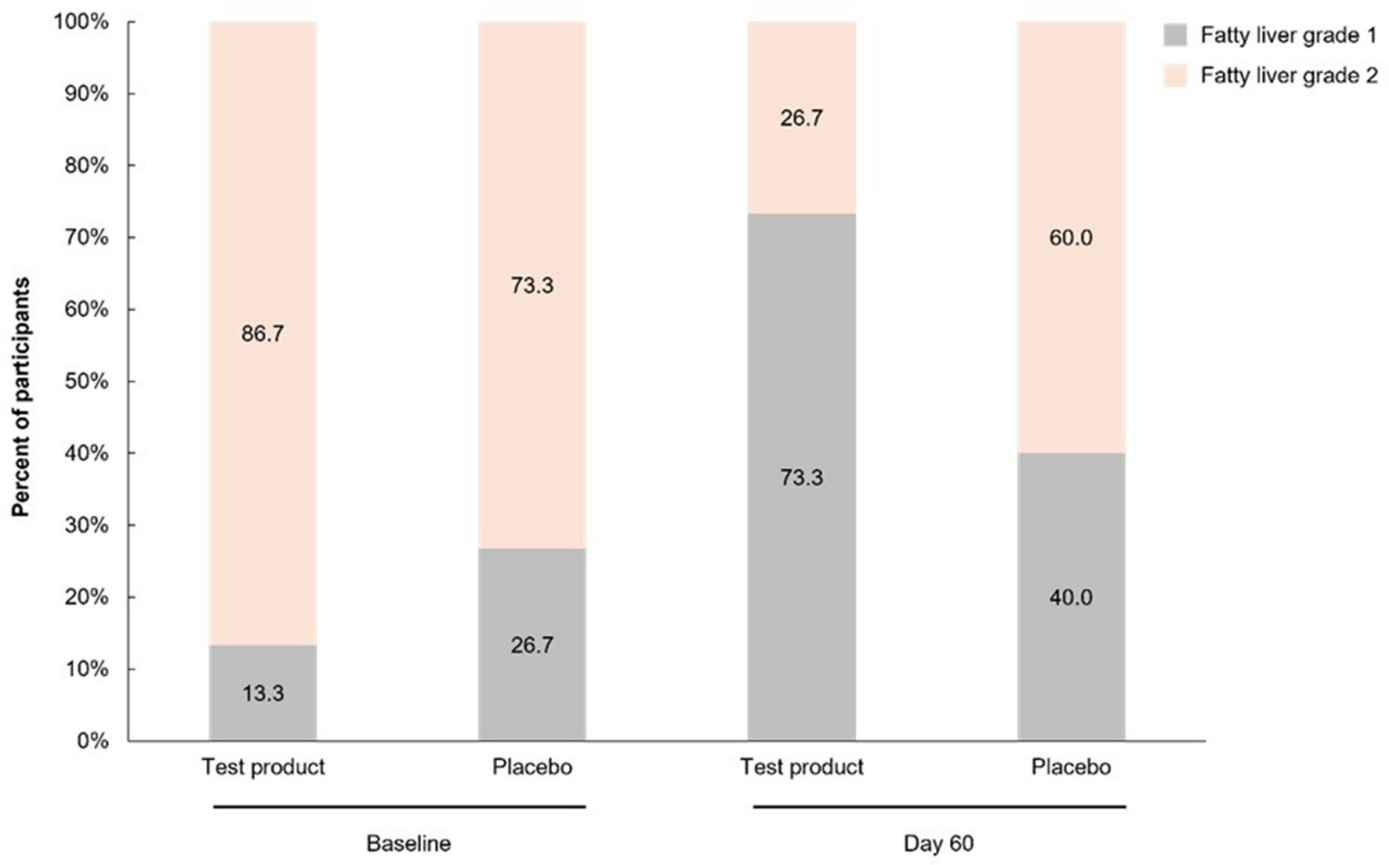
Percentage of participants with fatty liver grade 1 and grade 2 at baseline and on Day 60.

For the primary efficacy endpoint, a significantly greater percentage of participants in the test product group than placebo group had an improvement in fatty liver grading from baseline to Day 60 [60.0% (9/15) vs. 13.3% (2/15); *p* = 0.008] (Figure 2). There was a shift in the proportion of participants with grade 1 vs. grade 2 fatty liver from baseline to Day 60. In the test product group, grade 1 fatty liver was reported in 13.3% of patients at baseline and 73.3% at Day 60 and grade 2 fatty liver was reported in 86.7% at baseline and 26.7% at Day 60 (Figure 3). There were also less dramatic shifts in the placebo group, with grade 1 fatty liver reported for 26.7% at baseline and 40.0% at Day 60 and grade 2 fatty liver reported for 73.3% at baseline and 60.0% at Day 60.

Analysis of the secondary endpoints is shown in Table 2. Mean (SD) change from baseline to Day in fatty liver grading was significantly greater in the test product group compared with the placebo group [−0.60 (0.51) vs. −0.13 (0.35); *p* = 0.007], demonstrating a statistically greater reduction in steatosis grade. This was also demonstrated for the APRI score [−0.388 (0.122) vs. −0.104 (0.054), *p* = 0.0001) and CAP score [−27.4 (11.8) dB/m vs. −7.3 (4.8) dB/m, *p* = 0.0001]. The improvement in the lipid parameters of total cholesterol, HDL, and triglycerides from baseline to Day 60 was significantly greater in the test product group compared with the placebo group [total cholesterol: −7.7 (6.6) mg/dL vs. 0.7 (4.8) mg/dL, *p* = 0.0004; HDL: 1.9 (1.7) mg/dL vs. 0.0 (1.2), *p* = 0.001; triglycerides: −7.8 (8.0) mg/dL vs. −1.7 (4.4), *p* = 0.02]. There was not a significant difference between the groups in change from baseline to Day 60 in LDL values [−1.0 (7.6) mg/dL vs. −0.3 (2.3); *p* = 0.74]. Improvement in ALT and AST scores from baseline to Day 60 was also significantly greater in the test product vs. placebo group [ALT: −37.5 (5.1) IU/L vs. −9.5 (4.2) IU/L, *p* < 0.0001; AST: −30.9 (6.4) IU/L vs. −8.0 (2.5) IU/L, *p* < 0.0001].

**Table 2:** Change from baseline to day 60 in means of secondary endpoint parameters.

	**Test product (*N* = 15)**	**Placebo (*N* = 15)**	**Effect size of mean difference**	**95% Cl for mean difference**	***P*-value for mean difference**
Fatty liver grading	−0.60 (0.51)	−0.13 (0.35)	−1.05	[−0.80, −0.14]	0.007
APRI score	−0.388 (0.122)	−0.104 (0.054)	−2.93	[−0.36, −0.21]	0.0001
CAP score, dB/m	−27.4 (11.8)	−7.3 (4.8)	−2.17	[−26.8, −13.4]	0.0001
**Lipids, mg/dL**					
Total cholesterol	−7.7 (6.6)	0.7 (4.8)	−1.42	[−12.7, −4.1]	0.0004
HDL	1.9 (1.7)	0.0 (1.2)	1.26	[+0.80, +3.00]	0.001
LDL	−1.0 (7.6)	−0.3 (2.3)	−0.12	[−4.9, +3.5]	0.74
Triglycerides	−7.8 (8.0)	−1.7 (4.4)	−0.92	[−10.9, −1.3]	0.02
**Liver function test, mean (SD)**					
ALT, IU/L	−37.5 (5.1)	−9.5 (4.2)	−5.83	[−31.5, −24.5]	< 0.0001
AST, IU/L	−30.9 (6.4)	−8.0 (2.5)	−4.59	[−26.5, −19.3]	< 0.0001

Data are presented as mean (SD). Effect sizes are Hedges' *g*; *p*-values were calculated using two-sample *t*-tests. ALT, alanine transaminase; APRI, AST to platelet ratio index; AST, aspartate aminotransferase; CAP, controlled attenuation parameter; CI, confidence interval; HDL, high-density lipoprotein; LDL, low-density lipoprotein.

### 3.3 Safety

AEs were reported for 20.0% of participants (3/15) in the test product group and 26.7% of participants (4/15) in the placebo group, all of which were mild in severity and none were considered related to the study treatment (Table 3). In the test product group, one participant (6.7%) each had hyperchlorhydria, muscle spasm, and headache, while in the placebo group, one participant (6.7%) experienced a headache and four participants (26.7%) experienced pruritus. All AEs resolved within < 1 to 3 days with no change in study intervention dose and no study discontinuations; none of the AEs were considered to be causally related to the study intervention.

**Table 3:** Safety.

	**Test product (*N* = 15)**	**Placebo (*N* = 15)**
Any AE	3 (20.0)	4 (26.7)
**Preferred term**		
Hyperchlorhydria	1 (6.7)	0
Muscle spasm	1 (6.7)	0
Headache	1 (6.7)	1 (6.7)
Pruritus	0	4 (26.7)

Data are shown as *n* (%). Preferred terms were coded using the Medical Dictionary For Regulatory Activities v24.1. AE, adverse event.

## 4 Discussion

This phase 2 study of a nutraceutical liver support test product demonstrated significantly improved clinical and biochemical markers of MASLD after 60 days of twice daily treatment. These results suggest that this liquid nutraceutical formulation containing a mix of organic herbal extracts (turmeric, dandelion, ginger, and milk thistle) may exert hepatoprotective effects through antioxidant activity, modulation of lipid metabolism, or anti-inflammatory pathways, although mechanistic conclusions cannot be drawn from this study.

While not investigated in our study, the literature provides some insight into pathways that may be involved, at least in part, in the changes observed in our study. For example, curcumin is reported to support liver health by modulating hepatic lipid metabolism through the adenosine monophosphate-activated protein kinase (AMPK) and peroxisome proliferator-activated receptor alpha (PPAR-α) pathways while reducing oxidative stress via nuclear factor erythroid 2-related factor 2 (Nrf2) activation and suppressing inflammatory signaling through nuclear factor kappa-light-chain-enhancer of activated B cells (NF-κB) inhibition, mechanisms consistent with improvements seen in clinical MASLD populations (24). Silymarin, found in milk thistle, exerts hepatoprotective effects by enhancing antioxidant defenses, stabilizing mitochondrial function, attenuating inflammatory signaling through NF-κB modulation, and improving lipid metabolism and fibrotic pathways in liver disease models (25). Ginger contributes to liver support by decreasing inflammatory cytokines, improving oxidative stress balance, and enhancing insulin sensitivity and lipid metabolism, consistent with reductions in liver enzymes observed in patients with MASLD (26). Further, dandelion promotes hepatic protection by reducing hepatic lipid accumulation, improving β-oxidation and antioxidant activity, modulating inflammatory pathways, and influencing gut–liver axis dynamics in high-fat diet models (27). It is possible that the combination of these ingredients resulted in a synergistic clinical effect, though this was not evaluated our study. Future studies are needed to investigate the mechanisms behind the observed changes and to evaluate which specific ingredients may be responsible for these changes and whether the ingredients are acting synergistically.

The results are particularly relevant given the limited therapeutic landscape for MASLD/MASH. Resmetirom and semaglutide are the only U.S. Food & Drug Administration-approved medications available to treat fatty liver, and they are only approved for patients with severe disease (MASH) (28, 29). Clinical management of MASLD relies heavily on lifestyle modification—specifically, diet and exercise (3, 30). While lifestyle interventions remain essential, adherence and sustainability are challenging for many individuals, and the degree of improvement is often modest (3). Therefore, there is a clear unmet need for safe and effective interventions that can help address hepatic fat accumulation and inflammation in the early stages of MASLD and prior to the development of MASH. Further studies with a larger patient population will provide insight on whether the test product may be effective as an adjunct intervention alongside lifestyle modification in patients with MASLD.

In this study, 60% of participants had an improvement in fatty liver grading by one grade. This is clinically meaningful and promising given that it was observed 60 days after twice daily treatment. Although this result is encouraging, we acknowledge that fatty liver remained in all patients at the end of the study. Studies of a longer duration may help to clarify whether the test product would be able to further reduce fatty liver grading. Significant reductions in APRI and CAP scores were observed for the test product group compared with placebo (APRI, −0.388 vs. −0.104; CAP, −27.4 dB/m vs. −7.3 dB/m; *p* < 0.001 for both). Further, improvements were seen in total cholesterol, LDL, and triglycerides.

This trial is limited by its small sample size, short duration, lack of histological confirmation, and single-center design. Ultrasonography grading, while widely used, has inherent subjectivity. While participants were advised to maintain a simple, staple diet throughout the study, there were no strict dietary or exercise requirements, which may have confounded the observed effects of the test product. Further, unmeasured confounding factors, such as disease severity or metabolic comorbidities, may have impacted the results. Most study participants were male, potentially limiting the generalizability of the study findings to the female population. Future trials of a longer duration and with a larger sample size are needed to confirm these preliminary findings. Such studies should include similar non-invasive clinical tests and mechanistic biomarker analysis.

## 5 Conclusions

This liquid nutraceutical formulation containing a mix of organic herbal extracts (turmeric, dandelion, ginger, and milk thistle) demonstrated promising hepatoprotective activity in individuals with fatty and had an excellent safety and tolerability profile. The findings provide preliminary evidence supporting further evaluation of this nutraceutical in adequately powered multicenter, randomized trials using standardized quantitative imaging and biomarker outcomes.

## Data Availability

The raw data supporting the conclusions of this article will be made available by the authors, without undue reservation.

## References

[1] Wang D Miao J Zhang L Zhang L. Research advances in the diagnosis and treatment of MASLD/MASH. Ann Med. (2025) 57:2445780. doi: 10.1080/07853890.2024.2445780 41421798 PMC11703476

[2] Yoo JJ Yoo YJ Moon WR Kim SU Jeong SW Park HN . Correlation of the grade of hepatic steatosis between controlled attenuation parameter and ultrasound in patients with fatty liver: a multi-center retrospective cohort study. Korean J Intern Med. (2020) 35:1346–53. doi: 10.3904/kjim.2018.309 PMC7652655 31694366

[3] European European Association for the Study of the Liver European European Association for the Study of Diabetes European European Association for the Study of Obesity. EASL-EASD-EASO clinical practice guidelines on the management of metabolic dysfunction-associated steatotic liver disease (MASLD). J Hepatol. (2024) 81:492–542. doi: 10.1016/j.jhep.2024.04.031 38851997

[4] Younossi ZM Golabi P Paik JM Henry A Van Dongen C Henry L. The global epidemiology of nonalcoholic fatty liver disease (NAFLD) and nonalcoholic steatohepatitis (NASH): a systematic review. Hepatology. (2023) 77:1335–47. doi: 10.1097/HEP.0000000000000004 36626630 PMC10026948

[5] Antunes C Azadfard M Hoilat GJ Gupta M. Fatty Liver. Treasure Island (FL): StatPearls (2025). 28723021

[6] Rinella ME Neuschwander-Tetri BA Siddiqui MS Abdelmalek MF Caldwell S Barb D . AASLD practice guidance on the clinical assessment and management of non-alcoholic fatty liver disease. Hepatology. (2023) 77:1797–835. doi: 10.1097/HEP.0000000000000323 36727674 PMC10735173

[7] Jabczyk M Nowak J Hudzik B Zubelewicz-Szkodzinska B. Curcumin in metabolic health and disease. Nutrients. (2021) 13:4440. doi: 10.3390/nu13124440 34959992 PMC8706619

[8] Davaatseren M Hur HJ Yang HJ Hwang JT Park JH Kim HJ . Taraxacum official (dandelion) leaf extract alleviates high-fat diet-induced nonalcoholic fatty liver. Food Chem Toxicol. (2013) 58:30–6. doi: 10.1016/j.fct.2013.04.023 23603008

[9] Mohamed OI El-Nahas AF El-Sayed YS Ashry KM. Ginger extract modulates Pb-induced hepatic oxidative stress and expression of antioxidant gene transcripts in rat liver. Pharm Biol. (2016) 54:1164–72. doi: 10.3109/13880209.2015.1057651 26079851

[10] Alsahli MA Almatroodi SA Almatroudi A Khan AA Anwar S Almutary AG . 6-Gingerol, a major ingredient of ginger attenuates diethylnitrosamine-induced liver injury in rats through the modulation of oxidative stress and anti-inflammatory activity. Mediators Inflamm. (2021) 2021:6661937. doi: 10.1155/2021/6661937 33531877 PMC7837795

[11] Achufusi TGO Pellegrini MV Patel RK. Milk Thistle. Treasure Island, FL: StatPearls. (2025). 31082119

[12] Wei X Wang C Hao S Song H Yang L. The therapeutic effect of berberine in the treatment of nonalcoholic fatty liver disease: a meta-analysis. Evid Based Complement Alternat Med. (2016) 2016:3593951. doi: 10.1155/2016/3593951 27446224 PMC4947506

[13] Farsi F Mohammadshahi M Alavinejad P Rezazadeh A Zarei M Engali KA. Functions of coenzyme Q10 supplementation on liver enzymes, markers of systemic inflammation, and adipokines in patients affected by non-alcoholic fatty liver disease: a double-blind, placebo-controlled, randomized clinical trial. J Am Coll Nutr. (2016) 35:346–53. doi: 10.1080/07315724.2015.1021057 26156412

[14] Vrentzos E Ikonomidis I Pavlidis G Katogiannis K Korakas E Kountouri A . Six-month supplementation with high dose coenzyme Q10 improves liver steatosis, endothelial, vascular and myocardial function in patients with metabolic-dysfunction associated steatotic liver disease: a randomized double-blind, placebo-controlled trial. Cardiovasc Diabetol. (2024) 23:245. doi: 10.1186/s12933-024-02326-8 38987784 PMC11238408

[15] Abera M Suresh SB Malireddi A Boddeti S Noor K Ansar M . Vitamin E and non-alcoholic fatty liver disease: investigating the evidence through a systematic review. Cureus. (2024) 16:e72596. doi: 10.7759/cureus.72596 39610563 PMC11602675

[16] Chai C Chen L Deng MG Liang Y Liu F Nie JQ. Dietary choline intake and non-alcoholic fatty liver disease (NAFLD) in U.S. adults: national health and nutrition examination survey (NHANES) 2017-2018. Eur J Clin Nutr. (2023) 77:1160–6. doi: 10.1038/s41430-023-01336-1 37634048

[17] Sedhom SS El Wakeel LM Barakat EMF Shousha HI Shamkh MA Salama SH . The impact of choline supplementation on oxidative stress and clinical outcomes among patients with non-alcoholic fatty liver disease: a randomized controlled study. Ther Adv Chronic Dis. (2025) 16:20406223251358659. doi: 10.1177/20406223251358659 40838115 PMC12361734

[18] Sherriff JL O'Sullivan TA Properzi C Oddo JL Adams LA. Choline, its potential role in nonalcoholic fatty liver disease, and the case for human and bacterial genes. Adv Nutr. (2016) 7:5–13. doi: 10.3945/an.114.007955 26773011 PMC4717871

[19] Tian Y Zhou Y Liao W Xia J Hu Q Zhao Q . Flaxseed powder supplementation in non-alcoholic fatty liver disease: a randomized controlled clinical trial. Food Funct. (2025) 16:1389–406. doi: 10.1039/D4FO05847J 39878023

[20] Patel G Shah S D'Adamo CR. Long-term safety and efficacy of a highly purified plant-based nutraceutical for improving clinical parameters of liver function in healthy participants: a randomized, double-blind, placebo-controlled clinical trial. (2025) 12:1721748. doi: 10.1101/2025.09.26.25336688 PMC12875990 41659807

[21] Wang Y Gou Y Zhang L Li C Wang Z Liu Y . Levels and health risk of pesticide residues in Chinese herbal medicines. Front Pharmacol. (2021) 12:818268. doi: 10.3389/fphar.2021.818268 35177984 PMC8844025

[22] Lauffenburger JC Tesfaye H Solomon DH Antman EM Glynn RJ Lee SB . Investigating the ability to adhere to cardiometabolic medications with different properties: a retrospective cohort study of >500 000 patients in the USA. BMJ Open. (2023) 13:e075840. doi: 10.1136/bmjopen-2023-075840 PMC10649612 37949625

[23] Singh JA. Facilitators and barriers to adherence to urate-lowering therapy in African-Americans with gout: a qualitative study. Arthritis Res Ther. (2014) 16:R82. doi: 10.1186/ar4524 24678765 PMC4060486

[24] Vajdi M Hassanizadeh S Hassanizadeh R Bagherniya M. Curcumin supplementation effect on liver enzymes in patients with non-alcoholic fatty liver disease: a GRADE-assessed systematic review and dose-response meta-analysis of randomized controlled trials. Nutr Rev. (2025) 83:1–12. doi: 10.1093/nutrit/nuad166 38213188

[25] Abenavoli L Izzo AA Milic N Cicala C Santini A Capasso R. Milk thistle (Silybum marianum): a concise overview on its chemistry, pharmacological, and nutraceutical uses in liver diseases. Phytother Res. (2018) 32:2202–13. doi: 10.1002/ptr.6171 30080294

[26] Rahimlou M Yari Z Hekmatdoost A Alavian SM Keshavarz SA. Ginger supplementation in nonalcoholic fatty liver disease: a randomized, double-blind, placebo-controlled pilot study. Hepat Mon. (2016) 16:e34897. doi: 10.5812/hepatmon.34897 27110262 PMC4834197

[27] You Y Yoo S Yoon HG Park J Lee YH Kim S . *In vitro* and *in vivo* hepatoprotective effects of the aqueous extract from Taraxacum officinale (dandelion) root against alcohol-induced oxidative stress. Food Chem Toxicol. (2010) 48:1632–7. doi: 10.1016/j.fct.2010.03.037 20347918

[28] US Food and Drug Administration. FDA Approves Treatment for Serious Liver Disease Known as ‘MASH' (2025). Available online at: https://www.fda.gov/drugs/news-events-human-drugs/fda-approves-treatment-serious-liver-disease-known-mashhttps://www.fda.gov/drugs/news-events-human-drugs/fda-approves-treatment-serious-liver-disease-known-mash (Accessed November 7, 2025).

[29] US Food and Drug Administration. FDA Approves First Treatment for Patients With Liver Scarring Due to Fatty Liver Disease (2024). Available online at: https://www.fda.gov/news-events/press-announcements/fda-approves-first-treatment-patients-liver-scarring-due-fatty-liver-disease#:~:text=Information%20For,pressure%20and%20type%202%20diabetes (Accessed March 23, 2025).

[30] Younossi ZM Zelber-Sagi S Lazarus JV Wong VW Yilmaz Y Duseja A . Global consensus recommendations for metabolic dysfunction-associated steatotic liver disease and steatohepatitis. Gastroenterology. (2025) 169:1017–1032 e2. doi: 10.1053/j.gastro.2025.02.044 40222485

